# Supplementary material for: The BDNF Val66Met Polymorphism (rs6265) Modulates Inflammation and Neurodegeneration in the Early Phases of Multiple Sclerosis
Source: Genes (Basel). 2022 Feb 10;13(2):332. doi: 10.3390/genes13020332 (PMC8871843; doi:10.3390/genes13020332)
Supplement: Supplementary file 1 [file genes-13-00332-s001.zip › genes-1559425-supplementary.pdf]

Table S1. Variance in the first nine principal components in PCA.

PC	VE	PVE	CVE
1	6.59	0.24	0.24
2	5.31	0.20	0.44
3	2.79	0.10	0.54
4	1.77	0.07	0.61
5	1.35	0.05	0.66
6	1.20	0.04	0.70
7	0.96	0.04	0.74
8	0.84	0.03	0.77
9	0.74	0.03	0.80

Supplementary table legend: principal component (PC); Variance Explained (VE); Percentual Variance Explained (PVE); Cumulative Variance Explained (CVE).
